# High-efficiency acid–base catalysts: ZrO_2_, TiO_2_, amine, and Br functionalized porous polymers for CO_2_ and epoxide to cyclic carbonate conversion†

**DOI:** 10.1039/d5ra00392j

**Published:** 2025-03-10

**Authors:** Abbas A. Jawad, Sura A. Ahmed, Hasan J. Al-Abedi

**Affiliations:** a Midland Refineries Company MRC/AL Daura Refinery Company/Project Management Division Baghdad Iraq abbasajd5d@gmail.com abbasajd5d@outlook.com; b Department of Chemical and Biochemical Engineering, Missouri University of Science and Technology Rolla MO 65409-1230 USA; c Midland Refineries Company MRC/AL Daura Refinery Company/Maintenance Board Baghdad Iraq

## Abstract

The development of new materials capable of converting carbon dioxide (CO_2_) into value-added products has emerged as a crucial strategy in addressing global climate change and promoting sustainable industrial practices. As CO_2_ emissions continue to rise, innovative catalytic systems that facilitate its utilization as a C1 carbon source are gaining significant attention. Such advancements not only contribute to carbon capture and utilization (CCU) efforts but also support the transition toward greener chemical processes by reducing dependence on fossil-derived feedstocks. The design of high-performance heterogeneous catalysts with synergistic acid–base sites is particularly important for improving catalytic efficiency in CO_2_ conversion. In this study, bifunctional catalysts were synthesized by embedding ZrO_2_ and TiO_2_ (ZT) nanoparticles into a polyamide-imide polymer dope, followed by phase inversion using a “dry-jet, wet-quench spinning” process to form porous hollow fibers (PF). The fibers were then post-grafted with 3-aminopropyltrimethoxysilane (APF) to introduce amine functional groups and further modified with 1,2-dibromopropane at 110 °C to immobilize covalent hydrogen-bond donor groups (–OH and –NH) and nucleophilic (Br^−^) species. These heterogeneous bifunctional catalysts were then evaluated for the synthesis of cyclic carbonates from CO_2_ and epoxides. The catalytic performance was systematically investigated under various reaction conditions, including temperature, reaction time, solvent selection, and CO_2_ pressure. The optimized catalyst system achieved 100% styrene oxide (SO) conversion and >99% selectivity for styrene carbonate (SC) *via* the cycloaddition reaction using the Br@ZT–APF catalyst in the presence of solvents. Beyond achieving high conversion rates, the catalyst demonstrated excellent recovery, thermal stability, and recyclability for at least five consecutive cycles without significant loss of activity.

## Abstract 2

The development of new materials capable of converting carbon dioxide (CO_2_) into value-added products has emerged as a crucial strategy in addressing global climate change and promoting sustainable industrial practices.

## Graphical abstract

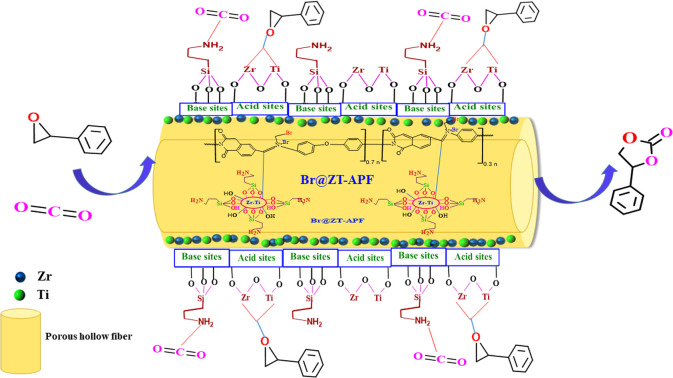

## 1. Introduction

CO_2_ capture and catalytic conversion are critical challenges that need to be addressed, as CO_2_ is the primary greenhouse gas. The development of advanced catalysts is highly desirable to enable the conversion of CO_2_ into high-value-added compounds under moderate conditions, given its thermodynamic stability and inert nature.^1^ In this regard, porous organic polymers provide an excellent platform for developing heterogeneous CO_2_ conversion catalysts due to their large surface areas, high thermal stability, diverse building blocks, and customizable porous structures. By modifying the frameworks of porous organic polymers, active sites can be incorporated to facilitate CO_2_ activation and conversion. This review highlights recent advancements in the design and synthesis of porous organic polymer-based heterogeneous catalysts for CO_2_ conversion. We primarily focus on the synthetic strategies employed to introduce active sites into porous organic polymer frameworks, including N-doping, metalation, and ionic functionalization. One of the most important CO_2_ fixation processes is the cycloaddition reaction, which converts epoxides and CO_2_ into five-membered cyclic carbonates. This process eliminates the need for hazardous phosgene while achieving 100% atom economy.^2,3^ Various catalysts have been reported for this reaction, including metal oxides,^4,5^ porphyrins,^6^ salen metal complexes,^7^ alkali metal salts,^8^ transition-metal complexes,^9^ quaternary ammonium or quaternary phosphonium salts,^10^ ionic liquids,^11^ organocatalysts,^12^ and MOFs.^13,14^ Among these, porous heterogeneous catalysts are in high demand and have gained significant attention. Cyclic carbonate derivatives are widely used due to their low toxicity, minimal odor, and high boiling points.^15^ Furthermore, they have applications in fuel additives, lithium-ion battery electrolytes,^16^ and polycyclic carbonate synthesis.^17^ Because CO_2_ is a kinetically inert molecule, its activation is crucial for efficient conversion. In this context, the direct coordination of a metal-doped hollow fiber CO_2_ complex is one of the most effective strategies for inducing chemical reactions with CO_2_. Numerous studies have explored different catalytic systems, as the production of cyclic carbonates from CO_2_ and epoxides aligns with the principles of sustainable development. To move toward practical applications, recent research has focused on an innovative class of efficient and porous heterogeneous systems – hollow fiber catalysts – for CO_2_ conversion and fixation. These systems offer advantages such as easy separation, recyclability, and non-leaching properties.^18^ Bifunctional catalysts, which contain two distinct catalytic sites, can enhance reaction rates through cooperative catalysis. Recent studies have demonstrated that organic amine molecules effectively catalyze the direct reaction between CO_2_ and epoxides to form cyclic carbonates.^19,20^ Various supported amines have been produced and studied for this reaction.^21,22^ Metal oxides, commonly used in heterogeneous catalysis, play a crucial role as both active phases and supports due to their acid–base and redox properties. Among these, the titania–zirconia combination has attracted considerable attention. Notably, zirconium dioxide (ZrO_2_) and titanium dioxide (TiO_2_) each exhibit excellent catalytic properties and have been employed as supports for various noble and transition metals in different catalytic applications. Combining two distinct oxides can introduce new physicochemical and catalytic properties by forming stable mixed oxide compounds. To the best of our knowledge, this is the first review to focus on the relevance and significance of mixed oxides in catalysis. Basing upon our previous work^23^ this study investigates the incorporation of ZrO_2_ and TiO_2_ (ZT) to porous hollow fibers (PF) crosslinked with 3-aminopropyltrimethoxysilane (A) and then alkylated with 1,2-dibromopropane to investigate cooperative Lewis acid/Brønsted acid sites (LAS/BAS) and Lewis base functionality and nucleophilic species suitable for use as heterogeneous catalysts in the production of styrene carbonate (SC) from CO_2_ and SO. Highly porous catalysts are created and extensively characterized using XRD, BET, FTIR spectrum, TGA, and pyridine IR analysis. Additionally, the effects of various experimental parameters such as reaction temperature, reaction time, CO_2_ pressure, and solvent selection are systematically investigated.

## 2. Experimental section

### 2.1. Chemicals

For the manufacture of polymer dope and the production of composite hollow fiber catalysts, the following compounds were used: zirconia (average particle size 100 nm, surface area of 600 m^2^ g^−1^, and pore size of 5 nm), titania (average particle size 100 nm, surface area of 41 m^2^ g^−1^, and pore size of 15 nm, and pore size of 4 nm) were purchased from Sigma-Aldrich. Torlon polyamide-imide, which is a commercially available (Solvay Advanced Polymers) (Alpharetta, GA), methanol (ACS grade, VWR), polyvinylpyrrolidone (with molecular weight = 300 from Sigma-Aldrich), *N*-methyl-2-pyrrolidone (NMP), *N*,*N*-dimethylformamide (DMF) (99%) (ACS Reagent, >99.8%, VWR), (Sigma-Aldrich, Reagent Plus, 99%, Milwaukee), and hexane (ACS Reagent, VWR, >98.5%). 3-Aminopropyltrimethoxysilane (assay = 95%), styrene oxide (SO) (assay = 97%), acetonitrile (ACN) (99.8%), DMF (assay = 99.8%), and 1,2-dibromopropane (97%) were obtained from Sigma Aldrich. The ultra-high pressure (UHP) N_2_ and CO_2_ gases were provided by Airgas.

### 2.2. Bromide-immobilized ZT–PF formation

**Scheme 1:**
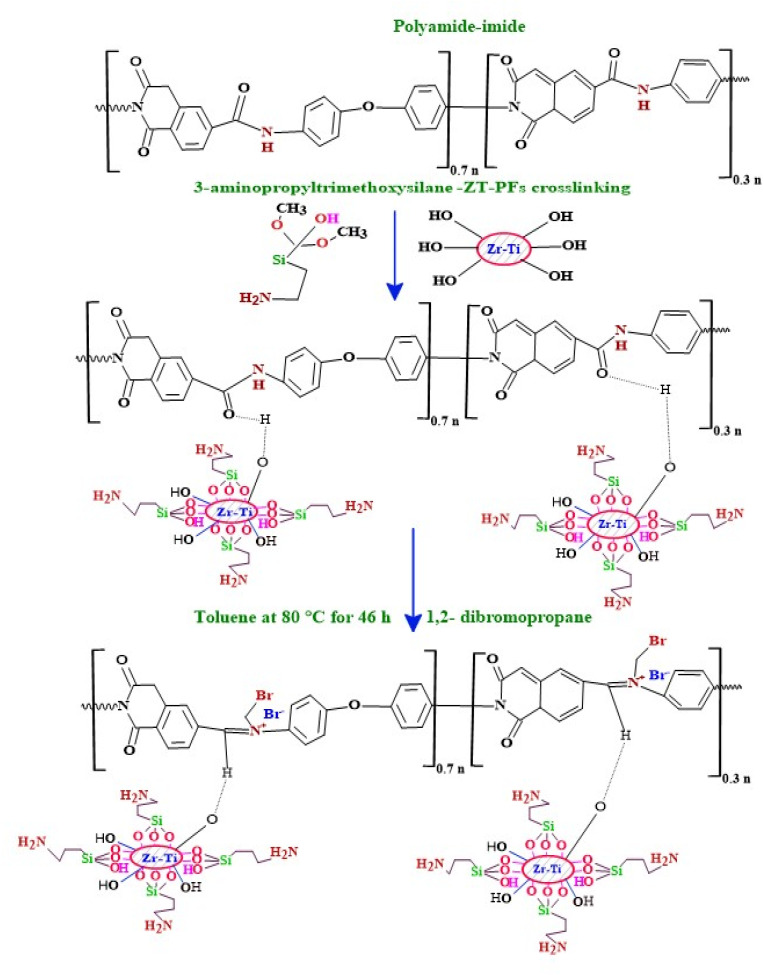
Illustrates Br@ZT–APFs crosslinking and alkylation of 3-aminopropyltrimethoxysilane-grafted PFs with 1,2-dibromopropane in dry toluene.

The “dry-jet, wet-quench spinning” process employed in this study to create ZrO_2_–TiO_2_ (ZT) with poly(amide-imide) hollow fibers (PFs) has been discussed in depth in earlier publications.^23^ Porous hollow fibers dissolve easily in polar aprotic solvents, hence they should be post-treated before being used as heterogeneous catalysts. To enhance the ability and compatibility of the hollow fibers in polar aprotic solvents (*e.g.*, ACN and DMF) that are commonly used in chemical transformation reactions in flow, the porous poly(amide-imide) hollow fibers were grafted with 3-aminopropyltrimethoxysilane (A) in organic network structures by further condensation of 3-aminopropyltrimethoxysilane at 80 °C for 2 h in a mixture of toluene and water. Ammonium bromide compounds were obtained by post-synthetically enhancing the ZT–APF with 1,2-dibromopropane in dry toluene at 80 °C for 4–6 h, denoted herein as Br@ZT–APF. The excess alkyl halide was then removed *via* solvent exchange with toluene, and the hood was left out overnight to dry. Finally, the fibers were vacuumed (30 mTorr) at 85 °C to release excess solvent from the pores as depicts in Scheme 1.

### 2.3. Catalyst characterization

Nitrogen physisorption isotherms were determined at 77 K using Micromeritics 3Flex surface characterization analyzer equipment. Prior to analysis, the hollow fibers were degassed for 2 h at 110 °C under vacuum. The Brunauer–Emmett–Teller (BET) and Barrett–Joyner–Halenda (BJH) techniques were used to determine surface area and pore size distribution.

Using a Bruker Tenser spectrophotometer, all post-treated fibers were evaluated *via* FTIR at room temperature in the range of 400–4000 cm^−1^ with a resolution of 4.0 cm^−1^.

The Br@ZT–APF composition was determined by bulk elemental analysis (ICP-MS) and CHN analyses (PerkinElmer Series II, 2400) were carried out to evaluated the concentration of TiO_2_ and ZrO_2_, nanoparticles, as well as amine and bromine loadings of the hollow fibers.

GC-MS (Hewlett Packard 5890E Series II Plus Gas Chromatograph) with a mass selective detector (Hewlett Packard/HP 5972 GC-MS System) was used to evaluate the reaction mixture as displayed in (see Fig. S2, ESI†).

The results were examined *via* the proton nuclear magnetic resonance (^1^H NMR) recorded on a 400 MHz Bruker Avance III employing CDCl_3_ as the solvent. Furthermore, the chemical shifts (*δ*), reported in parts per million (ppm), are relative to the residual CHCl_3_ peak (7.87 ppm for ^1^H NMR) (see Fig. S3, ESI†). The coupling constants (*J*) are reported in Hertz (Hz). ^13^C Magic Angle Spinning Nuclear Magnetic Resonance (MAS NMR) was recorded using solid-state NMR instrument (400 MHz Bruker Avance III) (see Fig. S4, ESI†).

Fourier-transform infrared spectroscopy (FTIR) of pyridine (Py-IR) was used with a Bruker Tensor spectrophotometer to identify the types of acid sites that were present in the samples in order to ascertain the nature of surface acid sites. For the purpose of pyridine adsorption, all samples were activated at 450 °C for four hours in order to remove moisture, and then they were cooled to 60 °C till saturation. Recalculated to a 10 mg “normalized” wafer, all observed spectra were obtained. Lewis acid site (LAS) and Brønsted acid site (BAS) were identified as the bands at 1450 and 1550 cm^−1^, respectively, for the purpose of quantitatively characterizing acid sites.

Thermogravimetric analysis (TGA) of the catalysts was carried out from 25 to 800 °C using TGA (Model Q500, TA Instruments), at a heating rate of 20 °C min^−1^ under nitrogen atmosphere. The flow rate was 60 mL min^−1^ under nitrogen atmospheres.

The surface topographies were assessed by high-performance field emission using scanning electron microscopy (SEM) on a Zeiss Merlin Gemini microscope.

### 2.4. Cycloaddition reactions

To carry out an example of an active catalyst catalytic process, 3.5 mL of styrene oxide, 10 mL of *N*,*N*-dimethylformamide (DMF) or acetonitrile (ACN) (used as the solvent), and a high-pressure autoclave (Parr, USA) with a magnetic stirrer and a jacket heater, 200 mg of the active catalyst was added to the reaction mix. After the reactor was sealed, it was carefully flushed three times with CO_2_ to remove O_2_, then pressurized to 5–40 bar with CO_2_ and heated with subsequent stirring at an operating temperature of 80–160 °C for 1–8 h. After completing the reaction, the temperature of the autoclave was slowly decreased to room temperature. Furthermore, the decompression was performed slowly for 30 minutes to reduce reaction mixture loss, after which the liquid was degassed. After depressurization, the autoclave was gently opened, and the reaction mixture was separated by filtration (Whatman, diameter 55 mm). The mixture was evaluated using gas chromatography (Varian 3800) with an Elite Wax capillary column (DB-5) and a flame ionization detector (FID).

## 3. Results and discussion

### 3.1. Catalyst characterization

#### 3.1.1. XRD analysis

**Fig. 1:**
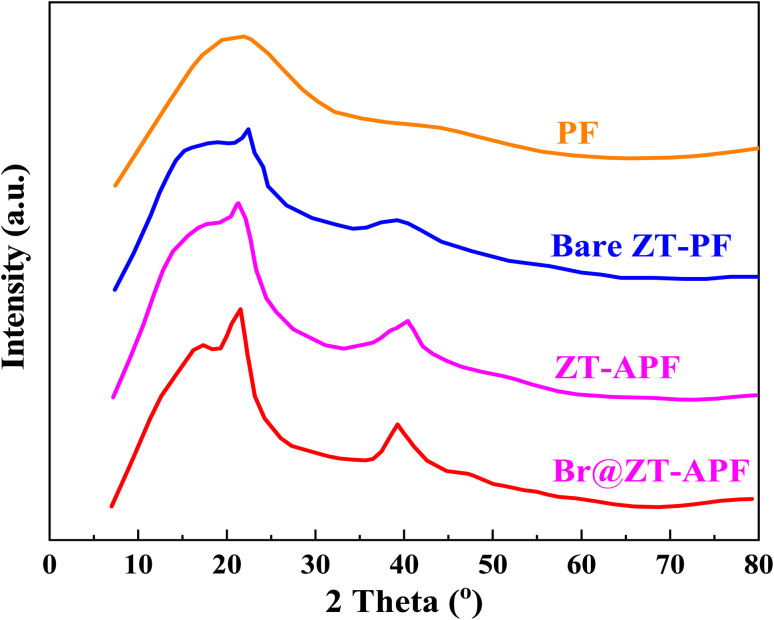
XRD patterns of the PF, bare ZT–PF, ZT–APF, and Br@ZT–APF.

XRD is a well-known method for characterizing organic–inorganic hybrids. Fig. 1 shows the XRD pattern of pure porous hollow fibers, with a peak at 2*θ* = 20° indicating an amorphous PF structure. Fig. 1 shows the XRD patterns for bare ZT–PF, ZT–APF, and Br@ZT–APF nanocomposites, respectively. The dispersion of metal oxides (ZrO_2_ and TiO_2_), 3-aminopropyltrimethoxysilane, and bromine into the porous hollow fibers matrix resulted in two diffraction peaks at 2*θ* of 22° and 40°, indicating a partial improvement over PF.

#### 3.1.2. Textural analysis

**Fig. 2:**
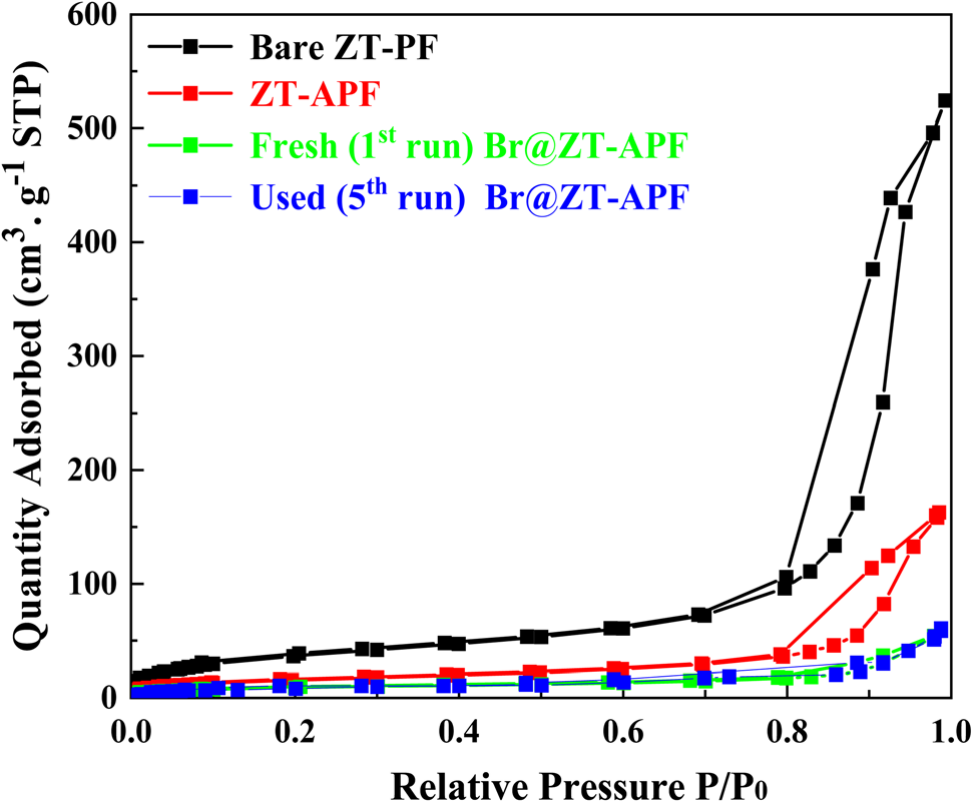
N_2_ isotherms of bare ZT–PF, ZT–APF, fresh (1st run) Br@ZT–APF, and used (5th run) Br@ZT–APF at 77 K.

**Fig. 3:**
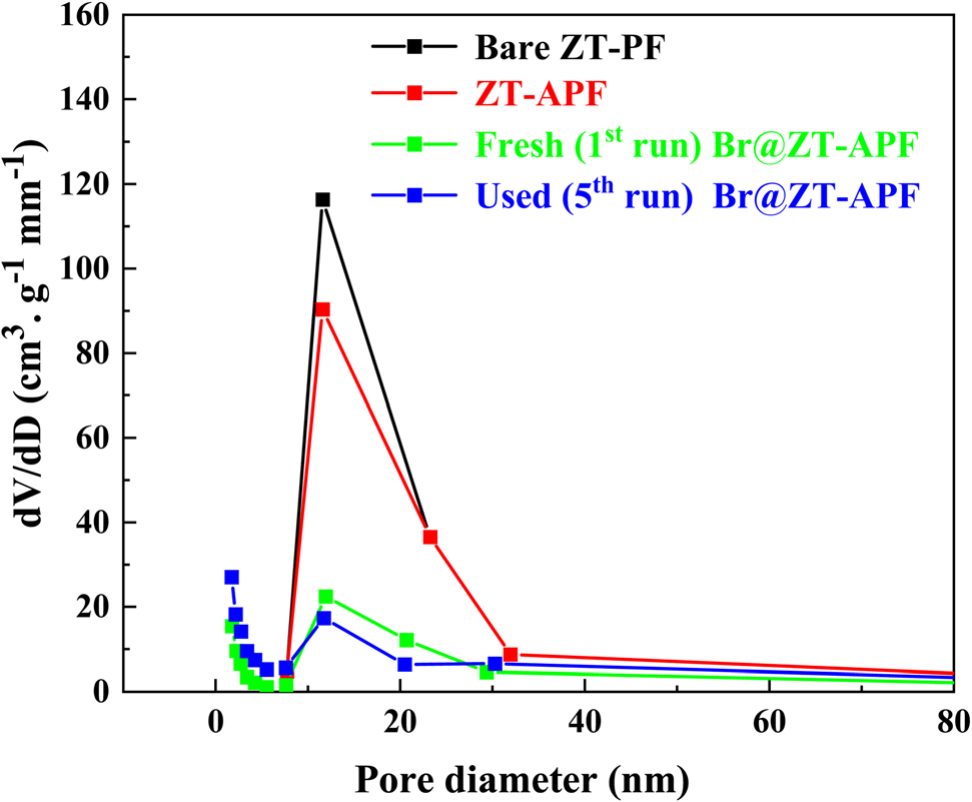
PSD of bare ZT–PF, ZT–APF, fresh (1st run) Br@ZT–APF, and used (5th run) Br@ZT–APF.

Textural properties of bare ZT–PF, ZT–APF, fresh (1st run) Br@ZT–APF, and used (5th run) Br@ZT–APF are summarized in Table 1. The N_2_ physisorption isotherms of the catalysts are depicted in Fig. 2. The incorporation of metal oxides, bromide, and aminosilane grafting resulted in a significant decrease in nitrogen uptake, indicating the successful incorporation of aminosilane moieties into the hollow fibers. This process partially filled the smaller pores within the corrugated fiber substructure, leading to a partial collapse of the pore network in the hollow fiber catalysts after aminosilane grafting. As observed in Table 1, the specific surface area and pore volume of Br@ZT–APF significantly decreased by approximately 66% following post-treatment modifications. This decline can be attributed to the anchoring of amine groups responsible for 3-aminopropyltrimethoxysilane crosslinking within the inner zirconia–titania surface and pore walls. Additionally, amine moieties likely adhered to the pores of the hollow fiber sorbent, while bromide immobilization within ZT–APF further contributed to the reduction in pore volume.^23^ This is consistent with XRD profile (Fig. 1). The adsorption–desorption curves presented in Fig. 2 exhibit non-overlapping behavior in the high-pressure region and display inflection points at lower relative pressures (*P*/*P*_o_ = 0.4–1.0). Furthermore, the catalysts exhibit type IV isotherms with an H3 hysteresis loop according to IUPAC classifications which are typical for materials with small mesopores.^24^ The presence of an inflection point in the nitrogen adsorption isotherm further corroborates the successful synthesis of mesoporous catalysts. Because of surface adsorption methods and the creation of mono and multilayers, the position of *P*/*P*_o_ at the inflection point is a function of pore diameter.^25^ At *P*/*P*_o_ = 0.6, nitrogen fills a small percentage of the pore volume in small pores, indicating the creation of a monolayer on micropore catalyst. Furthermore, with an increase in pressure (*P*/*P*_o_ = 0.6–0.9), the majority of the porous space with a larger size is filled with nitrogen, reflecting the contribution of mesoporous to catalysts during adsorption. Because of nitrogen capillary condensation in the mesopore, there is a significant inflection on the catalyst's isotherm in this region. Some researchers believe that this phenomena is a property of a material's pore uniformity.^26,27^ The increase in N_2_ uptake in the higher relative pressure (∼0.96) which suggests the presence of interconnected pores, comprising both primary and secondary pores within the porous framework. The existence of smaller pores within the catalyst structure has been linked to their role as pore necks or bridges, influencing the overall structural stability.^28^ At higher relative pressures, a plateau appears in the isotherm beyond the capillary condensation phase. This phenomenon arises due to multilayer nitrogen adsorption on the catalyst's exterior surface and within empty slits, indicating a relatively small external surface area. The desorption branch of the isotherm does not completely overlap with the adsorption branch, implying that nitrogen adsorption within the mesopores occurs irreversibly likely due to a delay in the onset of capillary condensation. Finally, at high pressures (*P*/*P*_o_ > 0.8), a vertical inflection is observed, corresponding to nitrogen entry into macropores within the catalyst structure. Because the pore was filled with 3-aminopropyltrimethoxysilane and bromide, the isotherm ZT–APF after loading was lower than before loading. This loading process had a significant influence on the ZT–PF pore because the inner pore was covered by 3-aminopropyltrimethoxysilane and bromide molecules, preventing nitrogen from filling the pore.^29–32^Fig. 3 displays the pore size distribution curves obtained *via* the Barrett–Joyner–Halenda (BJH) method, which revealed narrow distributions centered at approximately 11.58, 11.57, 11.41, and 11.40 nm, respectively. As stated previously, the pore diameter declines slightly after the post-treatment processes due to pore clogging caused by the incorporation of amine groups and bromide-immobilization into PF.^33^ The pore size distribution exhibited sharp peaks, which mainly appear in the mesoporous region (<50 nm), from which one can infer that the samples possess a mesoporous structure with good ordering and a uniform pore size distribution.^34^ As a result, one of the key strategies for an efficient carbon dioxide adsorption system is to immobilize both amino silane, and nucleophilic species inside the densely linked pore cell walls within the fibers. These results are in excellent agreement with our previous work.^23^ The absence of micropores may benefit the catalytic process since the rate of the reaction is dictated solely by the rate of reagent adsorption by active catalytic centers, the production of products, and their desorption. The absence of significant changes in the pore distribution of the catalyst after the reaction show that recycling behavior is extremely promising.^35,36^ To further investigate the structural stability of the catalysts, inductively coupled plasma mass spectrometry (ICP-MS) was conducted before and after post-modification and reaction. The results, as outlined in Table 1, indicate that the loadings of ZrO_2_, TiO_2_, nitrogen (amine), and bromine in the fiber bulk remained consistent before and after reaction, demonstrating that the chemical integrity of the fibers was preserved throughout the process. However, a slight decrease in bromide content was noted in Br@ZT–APF following the reaction, indicating minor leaching of bromide species.

**Table 1:** Amine loading and textural properties of the bare ZT–PF, ZT–APF, and post modified Br@ZT–APF (fresh and used reaction)

Materials	*S*_BET_ a [m^2^ g^−1^]	*V*_pore_ b [cm^3^ g^−1^]	TiO_2_ loading^c^ [wt%]	ZrO_2_ loading^c^ [wt%]	SiO_2_ loading^c^ [wt%]	Br loading^c^ [mmol g_fiber_^−1^]	N loading^c^ [mmol g_fiber_^−1^]
Bare-ZT–PF	57	0.25	3.0	10.0	10.0	—	—
ZT–APF	28	0.12	2.70	9.55	9.32	—	4.00
Br@ZT–APF (fresh 1st run)	24	0.068	2.55	9.32	9.26	0.25	3.56
Br@ZT–APF (used 5th run)	22	0.058	2.38	9.15	9.09	0.21	3.47

a Determined by N_2_ physisorption experiments at 77 K.

b Determined by BJH method.

c Determined by bulk elemental analysis (ICP-MS) and CHN analyses.

#### 3.1.3. Fourier transform infrared spectroscopy (FTIR) analysis

**Fig. 4:**
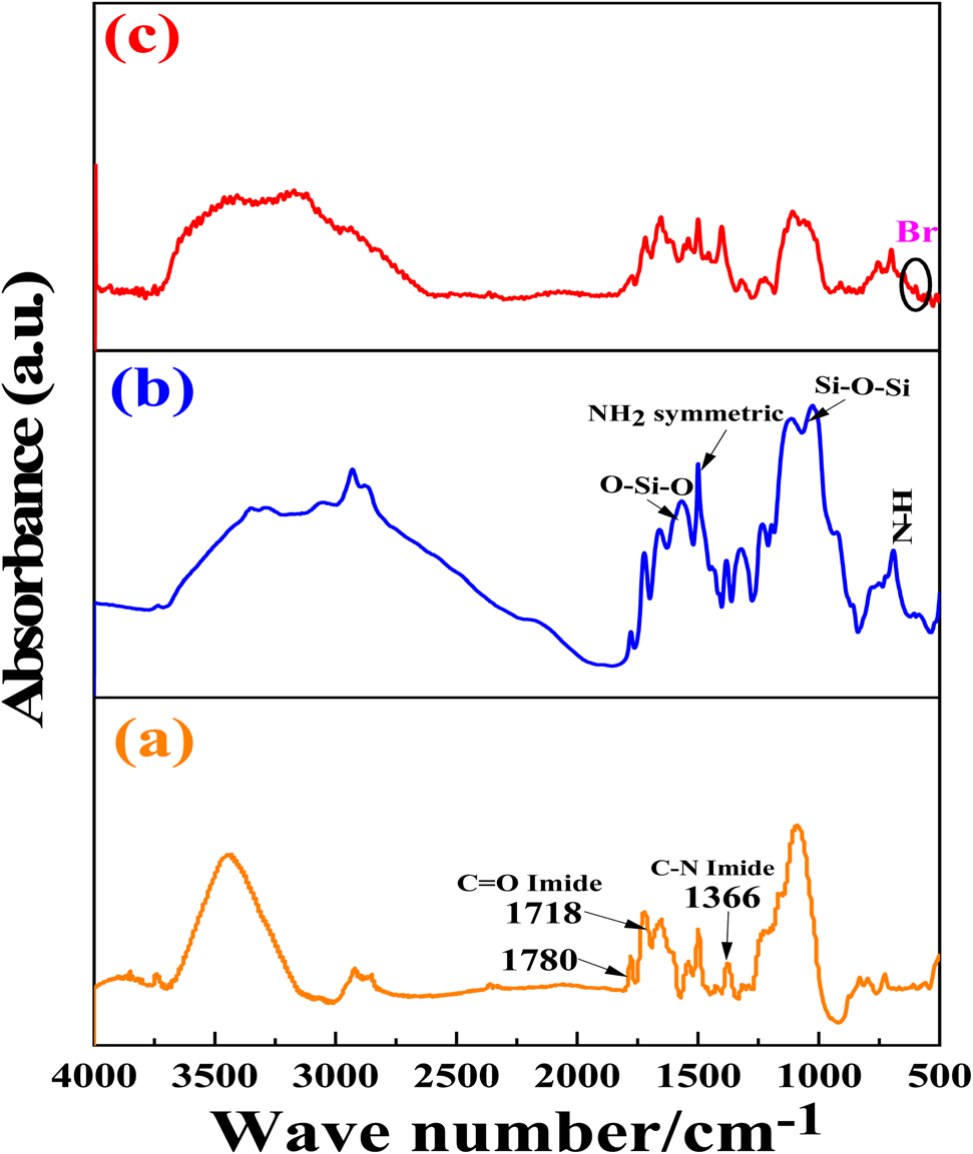
The FTIR spectra of (a) bare ZT–PF, (b) ZT–APF, and (c) Br@ZT–APF.

The successful bromide and 3-aminopropyltrimethoxysilane-grafting of hollow fiber catalysts was established by comparing their FTIR spectra before and after bromide and amine-grafting. Fig. 4 depicts the FTIR spectra in the 4000–500 cm^−1^ range of ZT–PF catalysts before and after bromide and aminosilane-grafting. The functionalized fibers showed a broad vibrational band in the range 3000–3500 cm^−1^, attributed to the O–H of hydrogen bonds in Si–OH and the HO–H of water molecules adsorbed on the catalyst surface. The stretching vibrations of the Si–O bonds usually appear at approximately *ν̄* = 1124 cm^−1^; therefore, the intense absorption band at approximately *ν̄* = 1055 cm^−1^ is due to the Si–O–Si bond asymmetric stretching.^23^ Furthermore, the peaks at ≈ 760–775 cm^−1^ and approximately *ν̄* = 505 cm^−1^ resulted from the existence of both tetragonal and monoclinic zirconia (Zr–O–Zr and Zr–O).^37–39^ Furthermore, the peak at approximately *ν̄* = 450–800 cm^−1^ is ascribed to the vibration mode of the (Ti–O–Ti and Ti–O bonds) and the peaks at *ν̄* = 1242, 1114, 1032, and 862 cm^−1^ correspond to the vibration mode of Ti–OH.^40^ This confirms that zirconia and titania were well dispersed in bare ZT–PF.^40^ The FTIR spectra of bare and amine-functionalized fibers were compared, and it was found that aminosilane grafting increased the intensity of vibrational bands vicinity ∼1000–1126 cm^−1^, 1618 cm^−1^, and 1489 cm^−1^ (which were attributed to Si–O–Si, O–Si–O, and N–H bending vibrations, respectively). This suggests that the condensation of 3-aminopropyltrimethoxysilane completed to form the Si–O–Si bond framework. The bare ZT–PF, ZT/APF, and Br@ZT–APF sorbents exhibit a strong imide asymmetrical carbonyl (C=O) absorbance at *ν̄* = 1780 cm^−1^, imide symmetrical carbonyl (C=O) absorbance at *ν̄* = 1718 cm^−1^, amide carbonyl (C=O) absorbance at *ν̄* = 1670 cm^−1^, amide N–C=O stretch at *ν̄* = 1500 cm^−1^, and imide C–N stretch at *ν̄* = 1366 cm^−1^. The spectra of bare ZT–PF, ZT–APF, and Br@ZT–APF sorbents exhibit absorbance peaks for amide functional groups, including a C=O stretch at *ν̄* = 1650 cm^−1^, a N–H stretch at *ν̄* = 1515–1570 cm^−1^, and a C–O–C stretch at *ν̄* = 1243 cm^−1^. Furthermore, the peaks at *ν̄* = 850 and 1270 cm^−1^ may be assigned to the Si–O and Si–CH_3_ stretching vibrations, respectively. The presence of a N–H bending vibration at *ν̄* = 688 cm^−1^ and a NH_2_ symmetric bending vibration at *ν̄* = 1489 cm^−1^ of the amine-grafted PF sorbents, absent in bare PF, indicates successful grafting of the aminosilane onto the surface (FP).^39^ There is a slight shift in the band position of the N–H bending vibration and of the NH_2_ symmetric bending vibration towards lower wavenumber in the case of the amine-grafted Zr–Ti/PF sorbents, and this may be due to bonding of zirconia and titania nanoparticles with the amine groups.^39^ As a result, the presence of 3-aminopropyltrimethoxysilane on the surface of ZrO_2_ and TiO_2_ was verified by the vibrational band at ≈ 3410–3230 cm^−1^ is related to N–H groups (from 3-aminopropyltrimethoxysilane and polymer backbone),^41^ indicating the presence of the imidazole ring. Alkyl C–H stretching bands were identified as the bands seen in the 2871–2926 cm^−1^ range. The band at ≈1616 cm^−1^ has been previously reported in the literature^42^ and is attributed to a conjugated C=N vibration of the cyclic system (imidazole) which is clearly seen in the cut-out of the IR spectra.^43^ The presence of the vibrational band at 596 cm^−1^ illustrated the functionalization of ZT–APF with 1,2-dibromoethane. The FTIR spectrum of the Br@ZT–APF confirmed the successfully 3-aminopropyltrimethoxysilane and bromide anchored on the surface of bare ZT–PF.

#### 3.1.4. Thermogravimetric analysis (TGA) analysis

**Fig. 5:**
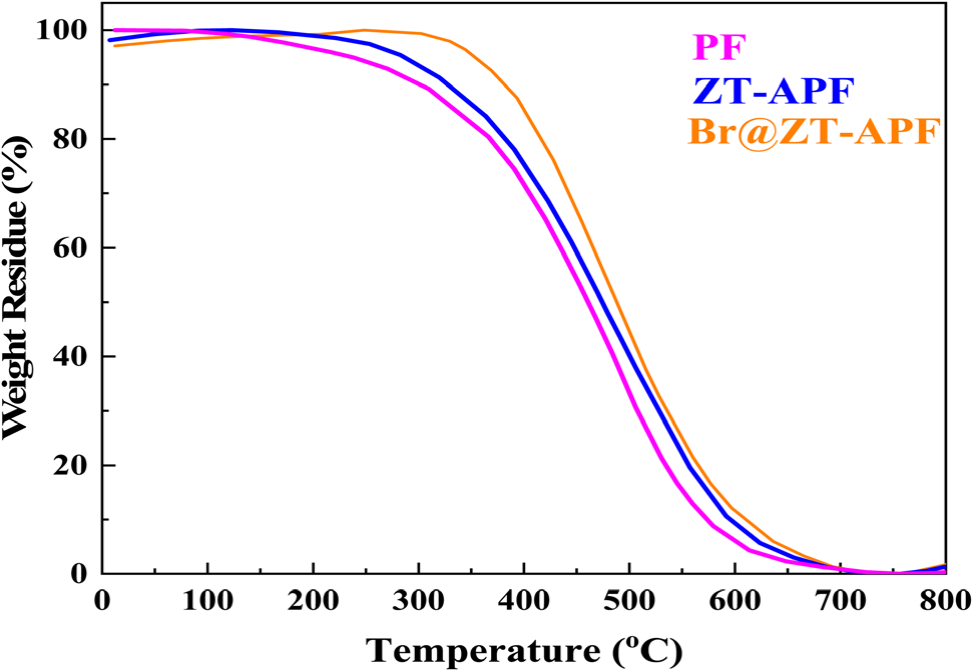
TGA plots for catalysts.

Thermogravimetric analysis (TGA) was used to investigate pure polyamide-imide, ZT–APF, and Br@ZT–APF nanocomposites, as seen in Fig. 5. The thermal stability of the polymer and nanocomposites was investigated using the *T*_onset_ and *T*_endset_ values of the samples and residue at 800 °C, as shown in Table 2. TGA curves and analytical findings in Table 2 show that the onset of decomposition temperature of the ZT–APF and Br@ZT–APF nanocomposites is greater than that of pure polyamide-imide at 300, 326, and 366 °C, respectively. Furthermore, the *T*_endset_ of nanocomposites greater than pure polyamide-imide are 581, 608, and 619 °C, respectively, as stated in Table 2. The nanoparticles clearly increase thermal stability due to the fine dispersion of transition metals, as well as amine and bromide, which results in a strong interfacial contact with the polyamide-imide matrix. Interfacial interaction plays a crucial role in the degradation of polymeric nanocomposites, *i.e.*, an excellent interfacial interaction allows particles to act as restriction sites for the mobility of a polymer chain, makes the scission of a polymer chain harder at lower temperatures, and thus shifts the material's degradation temperature to higher temperatures. Nonetheless, the deeper penetration of smaller particles in the polymer matrix will improve interfacial contact between additives and polymer chains, limiting polymer chain mobility. “Deeper penetration” refers to a further interaction between nano-scale particles and polymer chain segments, in addition to the simple interaction between nano-particles and polymer chains, resulting in improved thermal stability and excellent heat transport of nanocomposites that prevent heat collection at specific points. The degradation of the samples occurs in two stages, as is evident (Fig. 5). Weight loss in the first step is attributed to the removal of moisture and physisorbed water molecules, whereas weight loss in the second phase is connected to the polyamide-imide chain degradation process. At 800 °C, pure polyamide-imide, ZT–APF, and Br@ZT–APF nanocomposites have char yields (CR) of 55.6, 56.3, and 57.1%, respectively. The flame retardance of modified-PF will rise as its char yield increases. Furthermore, data shows that the samples treated with PF produced substantially more solid residues, and the char's surface was more compact. Heat is effectively prevented from reaching the substrate. Additionally, the Mars Van Krevelen and Hoftyzer equation is used to compute the limiting oxygen index (LOI) for materials (eqn (1)).^44^Table 2 displays the findings of limiting oxygen index.

1 LOI = 17.5 + 0.4 × CR

**Table 2:** Thermal properties of polyamide-imide and the nanocomposites

Catalysts	*T*_onset_ a (°C)	*T*_endset_ b (°C)	CR^c^ (%)	LOI^d^ (%)
Polyamide-imide	300	581	55.6	39.74
ZT–APF	326	608	56.3	40.02
Br@ZT–APF	366	619	57.1	40.34

a Degradation temperature.

b End of decomposition.

c Percentage weight of material left undecomposed after TGA analysis at maximum temperature 800 °C under nitrogen atmosphere.

d Limiting oxygen index (LOI) evaluating from char yield at 800 °C.

The LOI values for pure polyamide-imide, ZT–APF, and Br@ZT–APF nanocomposites are 39.74, 40.04, and 40.43 wt%, respectively, as determined from their char yield. Based on the LOI values, these materials can be categorized as self-extinguishing materials.^45^

#### 3.1.5. Scanning electron microscopy (SEM) analysis

**Fig. 6:**
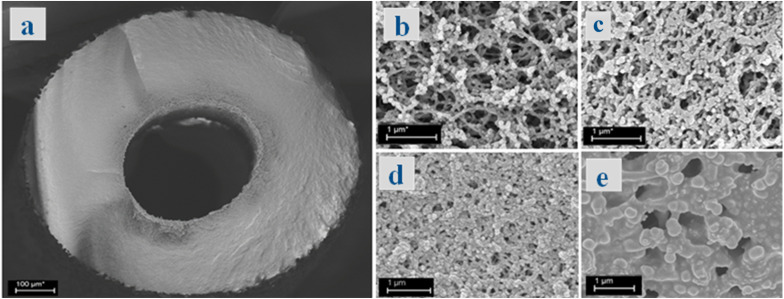
SEM images of (a) the cross section of bare ZT–PF; (b) surface of bare ZT–PF; (c) surface of ZT–APF; (d) surface of Br@ZT–APF (before reaction); and (e) surface of Br@ZT–APF (after reaction).

Fig. 6a and b depict SEM images of the cross-section and surface of bare ZT–PF. Fig. 6c and d depicts SEM images of the surfaces of aminosilane-grafted and Br-immobilized ZT–PF. The post-treatment of fibers may expand the polymer chains, resulting in a reduced pore size at the surface. Pore cells were observed to be smaller for Br@ZT–APF (Fig. 6d) compared to ZT–APF (Fig. 6c), resulting in a reduced surface area and pore volume. Furthermore, the longer post-treatment period and higher concentration of either 3-aminopropyltrimethoxysilane and 1,2-dibromopropane solutions had a detrimental effect on surface area and porosity. To enhance CO_2_ adsorption and conversion over Br@ZT–APF, concentration and post-treatment time parameters were modified accordingly. Moreover, as Fig. 6e makes evident, the fiber surface showed a moderate degree of porosity following the reaction in an ACN solvent, indicating both the swelling resistance of aminosilane-grafted polymeric hollow fibers and a strong link between the nucleophile and the polymer matrix. These micrographs qualitatively corroborate the preceding discussion of the fiber's textural qualities, showing that the fiber's outer pore shape collapsed during posttreatment processing.

### 3.2. Catalytic activity

#### 3.2.1. Effect of reaction temperature

Catalytic activity was positively impacted by raising the reaction temperature on SO conversion and SC selectivity over Br@ZT–APF. With the increment of temperature from 80 °C to 160 °C, the conversion of SO increased significantly from 90% to 100% as shown in Table 3. However, at higher reaction temperatures, the SO conversion leveled off. However, selectivity decreased slightly when the temperature was raised from 140 to 160 °C, possibly due to side reactions that occur at excessively high temperatures, such as isomerization and ring opening of SO, which initiate polymerization (SC) around the stronger acid sites, preventing further action. At increased temperatures, GC-MS investigations confirmed the presence of phenylacetaldehyde, benzaldehyde, acetophenone, and benzoic acid as a side reaction. The results revealed that the optimal temperature was 120 °C. Similar phenomenon was found in previous lituratures.^46–49^ Nonetheless, as demonstrated in Table 3, catalytic activity increased as temperature increased due to the thermal stability, better molecular mobility, and faster collision frequencies of the catalytic system at elevated temperature. It is important to notify that the catalyst's thermal durability is crucial for large-scale applications in exothermic processes, including the production of cyclic carbonates. Additionally, there was a noticeable improvement in the TOF with an increase in reaction temperature (Table 3). As the reaction temperature increased from 80 to 120 °C, the catalytic activity of Br@ZT–APF increased dramatically, resulting in a TOF increase from 4284 h^−1^ to 17 172 h^−1^.

**Table 3:** The effect of reaction temperature on the cycloaddition reaction with CO_2_ and SO^a^

Catalyst	Reaction temperature (°C)	Conversion (%) SO	Selectivity (%)					TOF^f^ (h^−1^)
			SC	PA^b^	BA^c^	ACP^d^	BZA^e^	
Br@ZT–APF	80	90	23	45	17	8	7	4284.0
	100	94	94	5	1	—	—	16 740
	120	100	99	1	0	—	—	17 172
	140	100	98	2	0	0	0	17 100
	160	100	98	2	0	0	0	17 100

a Reaction conditions: catalyst = 200 mg; styrene oxide = 3.5 mL; ACN (solvent) = 10 mL; CO_2_ pressure = 10 bar; reaction time = 4 h; temperature = 80, 100, 120, 140, and 160 °C.

b Phenylacetaldehyde.

c Benzaldehyde.

d Acetophenone.

e Benzoic acid.

f Turnover frequency: moles of quaternary ammonium ion per mol of SC product per h.

#### 3.2.2. Effect of CO_2_ pressure

**Fig. 7:**
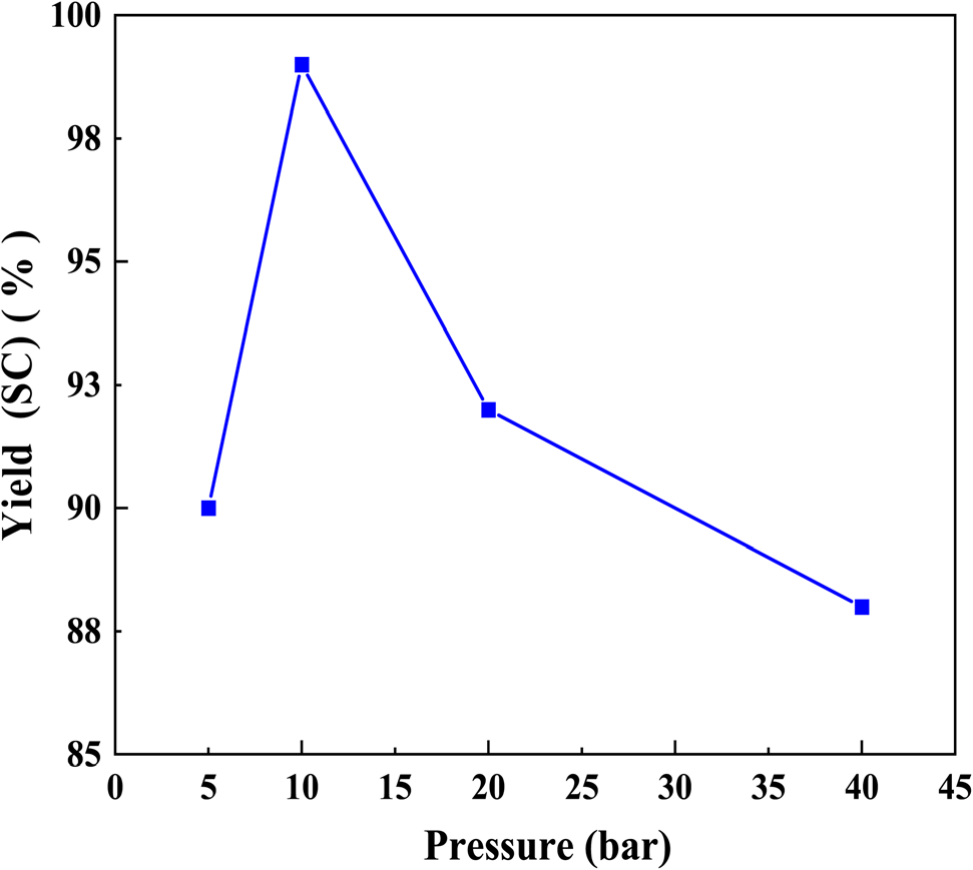
Effect of CO_2_ pressure on SC yield over Br@ZT–APF. Reaction conditions: catalyst mass = 200 mg temperature = 120 °C: reaction time = 4 h: amount of styrene oxide = 3.5 mL: amount ACN (10 mL) as solvent.

The effect of CO_2_ pressure on the cyclic carbonate yield in the presence of Br@ZT–APF is shown in Fig. 7. It is evident that pressure has a significant impact on the yield of cyclic carbonate. At lower pressures (5 bar), the SC yield of 90% can be achieved; it rises as CO_2_ pressure increases from 5 to 10 bar. When the reaction is carried out at 10 bar CO_2_, the SC yield can reach 99%. But as the pressure is raised to 40 bar, the SC yield dramatically drops to 88%. The effect of pressure on the CO_2_ and epoxide concentrations in the two phases explains this.^23^ The epoxide-rich phase is at the bottom and the CO_2_-rich phase is at the top. Because the catalyst is distributed in the liquid phase, this phase is where the reactions mostly occur. The substrate's partition behavior is correlated with changes in reaction rate, and the reactions are affected in two different ways by an increase in CO_2_ pressure. First, since CO_2_ is a reactant, its solubility in the epoxides increases as pressure rises, favoring the reaction. However, the concentration of epoxides in the liquid phase is too low and more epoxides are in the CO_2_-rich phase at higher pressures, which lowers the reaction rate, because CO_2_ functions as a solvent in the system and is regarded as a dense gas. At lower CO_2_ pressure, the first factor is dominant because the concentration of CO_2_ in the liquid phase is lower. The second factor becomes dominant when the pressure of CO_2_ is high enough. The optimum parameters for CO_2_ conversion and selectivity to SC were found to be 10 bar of pressure, 120 °C of reaction temperature, and 4 h of reaction time, consistent with the results of our previous work.^23^

#### 3.2.3. Effect of reaction time

Table 4 depicts the relationship between SO conversion, SC selectivity, and reaction time. SC selectivity rose rapidly with increasing reaction time before 2 h, then the increase trend slowed down and the highest conversion was obtained from 4 h to 6 h. Prolonged reactions beyond 6 h resulted in the decrease of SC selectivity and SO conversion slightly. It appears that the produced SC increased the viscosity of the reaction system, obstructing the interaction between catalyst and reactants during a longer reaction time. As a result, the optimal reaction time was determined to be 4 h.^42^

**Table 4:** The effect of time reaction on the cycloaddition reaction^a^

Catalyst	Solvents	Reaction time (h)	Conversion (SO) (%)	Selectivity (%)		
				SC	BA	PA
Br@ZT–APF	ACN	1	88	30	20	50
		2	95	50	33	17
		4	100	99	0	1
		6	100	99	0	1
		8	99	98	1	1

a Reaction conditions: catalyst = 200 mg; styrene oxide = 3.5 mL; acetonitrile (solvent) = 10 mL; CO_2_ pressure = 10 bar; temperature = 120 °C; reaction time = 1, 2, 4, 6, and 8 h.

#### 3.2.4. Effect of solvents

The effect of solvents on the cycloaddition reaction was researched under optimal condition. Table 5 presents the tabulated results, polar aprotic solvents (*i.e. N*,*N*-dimethylformamide (DMF) and acetonitrile (ACN)) provided different levels of selectivity and conversion. At 4 h with using the DMF as solvent, the SO conversion and SC selectivity both decreased (to 94% and 89%, respectively). In comparison to the acetonitrile solvent, the higher adsorption of the additional DMF solvent molecules on the catalyst's active sites may be strong with metal oxides, potentially deactivating certain catalyst active sites.^42,50,51^ In the context of this investigation, acetonitrile proved to be the most effective solvent for transferring the reactants to the catalyst's reactive site, as well as the high polarity of ACN facilitating the ability to stabilize intermediates, increase the efficiency between CO_2_ and styrene oxide, and enhance CO_2_ solubility, thereby leading to faster ring-opening of the styrene oxide. Results demonstrating that ACN solvent shifts the reaction toward the production of a desired cyclic carbonate (SC) and prevents its decomposition.^23,52^ In addition to acting as a solvent, it acts a dehydrating agent to remove water.^53^ As can be seen in Table 5, the effect of reaction time on SC selectivity over Br@ZT–APF in presence of ACN and DMF solvents is clear; longer reaction times resulted in a major drop in the process's chemo-selectivity, with larger levels of BA and PA produced. Whereas PA is created *via* Meinwald rearrangement, BA can be made by oxidative cleavage (which should result from diol precursors that might occur from water-induced ring opening of an epoxide or carbonate hydrolysis).^23^ This is in agreement with previous observations.^23,54,55^ Remarkably, in the acetonitrile condition, 100% conversion with 99% selectivity towards SC was observed within 4 h as shown in Table 5.

**Table 5:** The effect of solvents on the cycloaddition reaction^a^

Catalysts	Solvents	Reaction time (h)	Conversion SO (%)	Selectivity (%)		
				SC	BA	PA
Br@ZT–APF	ACN	1	88	30	20	50
		2	95	50	33	17
		4	100	99	0	1
		6	100	99	0	1
		8	99	98	1	1
Br@ZT–APF	DMF	1	83	11	20	80
		2	88	27	16	65
		4	94	89	3	8
		6	92	91	4	5
		8	92	91	4	5

a Reaction conditions: catalyst = 200 mg; styrene oxide = 3.5 mL; solvents (ACN, and DMF) = 10 mL; CO_2_ pressure = 10 bar; temperature = 120 °C; reaction time = 1, 2, 4, 6, and 8 h.

### 3.3. Catalyst reusability and product identification

**Fig. 8:**
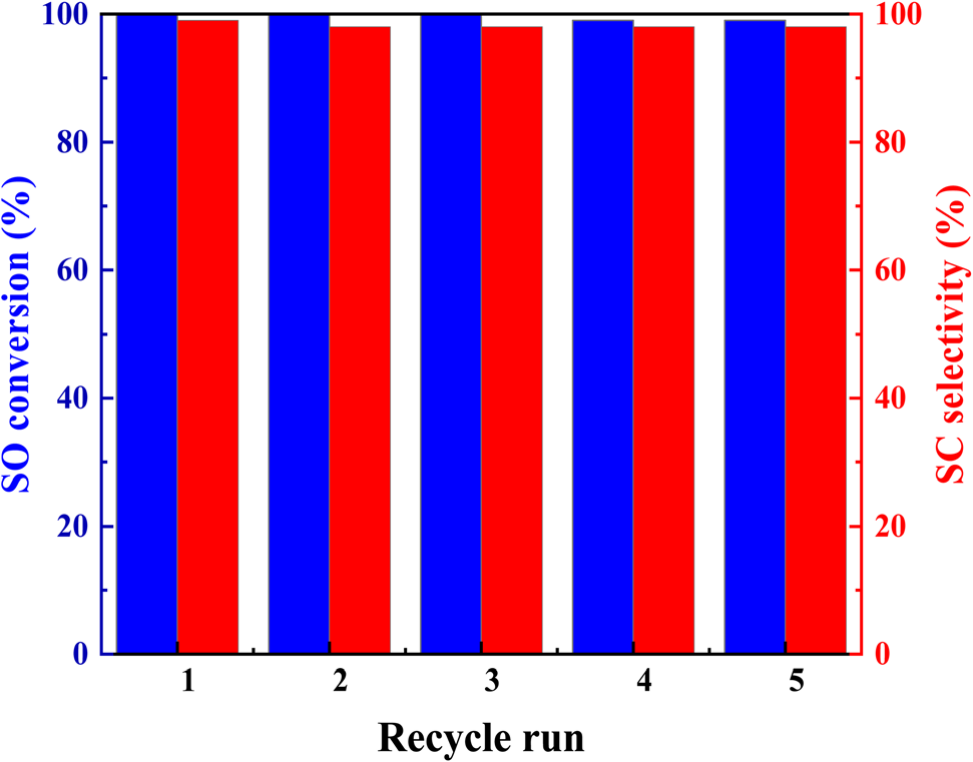
Catalyst recyclability study for cycloaddition reaction over Br@ZT–APF. Reaction conditions; catalyst mass = 200 mg; CO_2_ pressure = 10 bar; temperature = 120 °C; reaction time = 4 h; amount of styrene = 3.5 mL; amount ACN (10 mL) as solvent.

Recycling experiments were conducted to assess the reusability of the Br@ZT–APF catalyst. After each catalytic cycle, the catalyst was separated from the reaction mixture through simple filtration, followed by multiple washes with chloroform and acetone. To ensure complete removal of adsorbed reactants and products from the pores, the catalyst was reactivated under high vacuum at 110 °C before the subsequent cycle. Reusability studies indicated that Br@ZT–APF retained its catalytic activity over five consecutive cycles, with no significant decline in performance (see Fig. 8). A slight decrease in conversion efficiency was attributed to the partial loss of bromide ions and restricted accessibility to some active sites within the porous hollow fibers, as well as catalyst leaching due to fiber swelling.^56^ Nevertheless, the selectivity toward styrene carbonate (SC) remained stable at approximately 98.0%. The bromide loading in the reused catalyst was measured at 0.25 mmol g^−1^, which closely matched that of the fresh catalyst (0.21 mmol g^−1^), as confirmed by elemental analysis (ICP-MS) and CHN analysis (Table 1). To obtain a clear solution, the reaction mixture was diluted with acetonitrile and filtered. Upon solidification into a yellow crystalline substance, the mixture was subjected to column chromatography using silica gel. The isolated product was characterized using FT-IR, GC-MS, and NMR spectroscopy, which confirmed the formation of a colorless crystalline solid. The FT-IR spectrum (Nujol, cm^−1^) displayed characteristic absorption bands at 1802, 1380, 1344, 1166, 1090, 1155, 1015, 963, 937, 748, and 670 cm^−1^, corresponding to SC synthesis (Fig. S1, ESI†). The absorption peak at 1802 cm^−1^ was attributed to the stretching vibration of C=O, which corresponds to carbonyl stretch of SC (Fig. S1†). Similarly, increased intensities at 1344 and 1380 cm^−1^ confirmed asymmetric *ν*(C–O) stretching vibrations of the cyclic carbonate. The rate of epoxy group consumption correlated directly with cyclic carbonate formation,^46^ as evidenced by the diminishing intensities of epoxide-related peaks (C–O) at 815 and 876 cm^−1^ for styrene oxide (SO).^57^ Throughout the recycling experiments, the structural integrity of the catalyst remained intact, as demonstrated by the consistency in peak characteristics between the fresh and 5th run catalysts. This demonstrates the synthetic catalyst's stability, reusability, and efficiency. These bands were consistent with those of SC, as validated by an analysis of its GC-MS spectra. The GC-MS spectrum showed the molecular ion peak at *m*/*z* 164 (M)^+^ which corresponds to C_9_H_8_O_3_ (see Fig. S2, ESI†). Other fragments were at *m*/*z* 119, 105, 91, 90 (base peak), 78 and 65. ^1^H NMR (400 MHz, CDCl_3_), *δ* (pmm): 8.15–7.87 (m, 5H, AR), 6.392 (t, 1H, 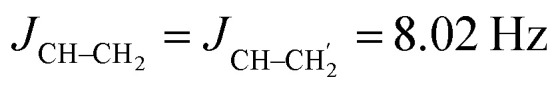), 5.466 (t, 1H, CH_2_, 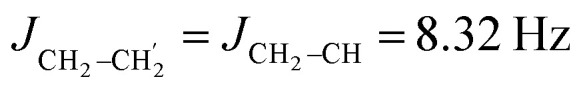), 4.995 (t, lH, 
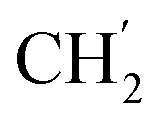
, 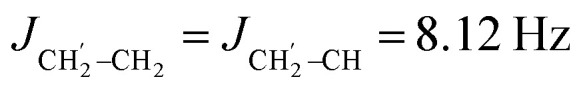) as shown in Fig. S3, ESI.† This finding is consistent with MS-GC profile (see Fig. S2, ESI†). ^13^C NMR (400 MHz, CDCL_3_), *δ* (ppm):155.98 (OCOO), 136.08 (AR_*ipso*_), 129.58 (AR_*meta*_), 127.08 (AR_*para*_), 126.13 (AR_*ortho*_), 79.61 (CH), 72.04 (CH_2_), respectively (see Fig. S4, ESI†). Spectroscopic data were identical to those found in previous literature reports for this particular chemical species.^46,58^ Also, the characterization results of ^1^H NMR and ^13^C NMR confirmed the structure of SC.

### 3.4. Characterization analysis of reused catalyst

#### 3.4.1. Infrared spectroscopy of pyridine adsorption (Py-IR)

**Fig. 9:**
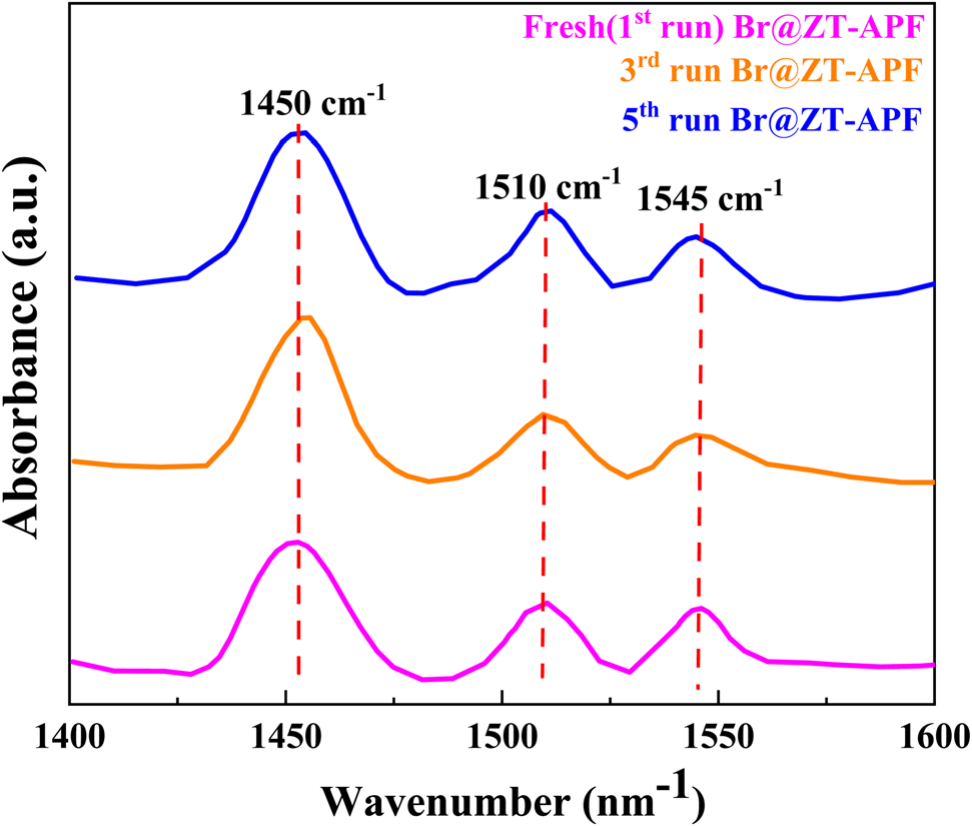
Pyridine IR spectra of Br@ZT–APF catalyst after evacuation at 150 °C.

*Ex situ* (FTIR) spectroscopy of pyridine adsorption was employed to assess the Lewis acid sites (LAS) and Brønsted acid sites (BAS) present on the catalyst surface. The analysis was conducted using a Bruker Tensor spectrophotometer, with transmission FTIR spectra providing insights into the catalytic activity of Br@ZT–APF (Fig. 9 and Table 6). To perform the analysis, one milliliter of dry pyridine was adsorbed onto 30 mg of catalyst for 14 hours. The catalyst was subsequently subjected to a drying process at 150 °C for one hour to remove loosely adsorbed pyridine. The dried sample (10 mg) was then homogenized with 200 mg of dry potassium bromide (KBr), followed by pelletization under hydraulic pressure to ensure uniformity. The FTIR spectra of these pellets were then recorded. As shown in Fig. 9, the pyridine adsorption bands provided quantitative insights into the LAS and BAS present in the metal oxide-doped hollow fiber catalysts. The absorption band observed at 1445 cm^−1^ was attributed to pyridine bound to LAS, whereas the intense band at 1510 cm^−1^ corresponded to pyridine chemisorbed on both LAS and BAS.^59,60^ Additionally, the band at 1545 cm^−1^ was indicative of pyridine adsorption on BAS.^59^ The acid site concentrations at 1450 and 1545 cm^−1^ were determined using the integrated molar extinction coefficients reported by Emeis,^60^ as detailed in Table 6. The presence of LAS and BAS was confirmed across all catalyst samples, including the fresh, third-run, and fifth-run catalysts, suggesting successful functionalization of the catalyst. However, a slight decrease in band intensity was observed after multiple reuse cycles (third and fifth runs), indicating strong interactions between the adsorbed pyridine and the Ti^4+^–Zr^4+^ species in the fibers (Table 6).^61–63^ These findings demonstrate that the Br@ZT–APF catalyst maintains a sufficient concentration of LAS and BAS, which contributes to its cooperative catalytic behavior.^60,64^ The coexistence of adequate LAS and BAS is particularly advantageous, as it facilitates the activation of epoxide molecules through coordination of the oxygen within the three-membered ring structure. This activation enhances the nucleophilic attack of bromide ions, promoting the efficient ring-opening of the epoxide and driving the catalytic reaction forward.^65–67^

**Table 6:** Amount of acid sites determined from pyridine-FTIR data

Catalyst	Brønsted acidity^a^ (μmol g^−1^)	Lewis acidity^a^ (μmol g^−1^)	Catalytic cycle
Br@ZT–APF	12.0	30.0	Fresh (after 1st run)
	11.0	27.84	Used (after 3rd run)
	9.54	24.21	Used (after 5th run)

a The amount of acidity for BAS and LAS was determined by Emeis.^68^*C*(pyridine on Brønsted acid sites) = 1.88IA(B)*R*^2^/*W*, *C*(pyridine on Lewis acid sites) = 1.42IA(L)*R*^2^/*W* (*R* = 13 mm, *W* = 30 mg).

#### 3.4.2. Textural analysis and elemental analysis (ICP-MS) and CHN analyses

The textural analysis of the fresh and recycled catalyst (after the 5th run) was very similar (Table 1 and Fig. 2, 3). This means that the morphology of the catalyst, the mesoporous micro/mesoporous structure is very robust after the reaction. Additionally, elemental analysis (ICP-MS) and CHN analyses of fresh and reused Br@ZT–APF nanocatalyst were investigated and results are listed in Table 1. The result of elemental analysis of reused catalyst displayed remains unaltered in loading contents of metal oxides.

### 3.5. Proposed mechanism of cyclic carbonate synthesis from styrene oxide and CO_2_

**Scheme 2:**
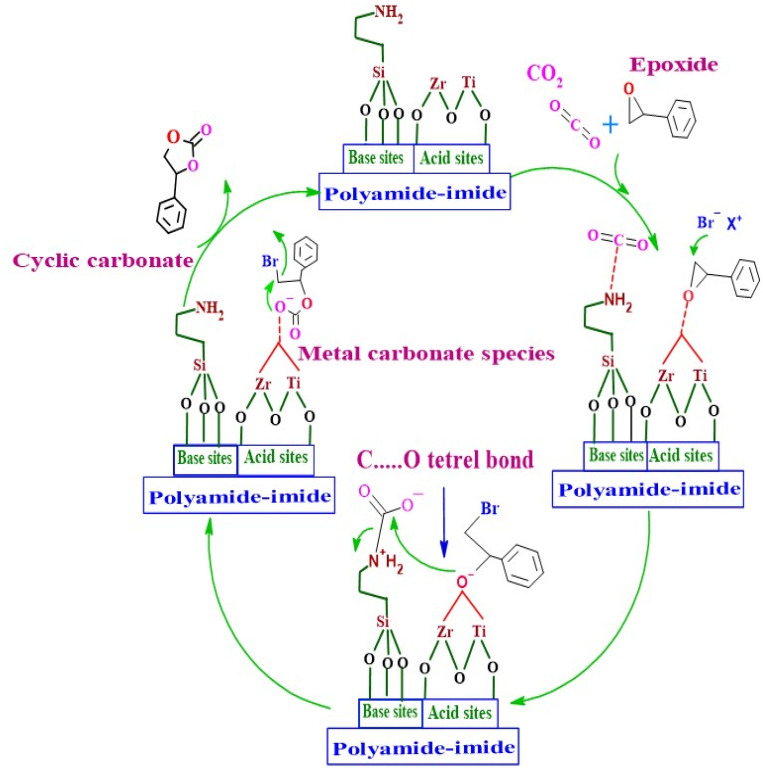
The proposed reaction mechanism for cycloaddition of epoxide and CO_2_, as catalyzed by Br@ZT–APF.

In this study, the polyamide imide hollow fibers are functionalized using aminosilanes and a bromine source to immobilize nucleophilic [Br^−^] species and covalently hydrogen-bond donor groups (–OH and –NH) on the porous hollow fiber surface. Highly efficient acid–base-nucleophilic producing trifunctional catalysts as shown in Scheme 2. The cycloaddition reaction is initiated by coordination of the oxygen atom of the epoxide with unsaturated Lewis acidic (ZrO and TiO) centers present in the larger channel, which activates the epoxy ring, the Lewis acid plays a crucial role in activating the epoxide by interacting an electrophilic attack on one of the oxygen atoms (SO). At the same time, the Br^−^ anion, generated from ammonium salt (polyamide-imide backbone structure) nucleophilically attacks the sterically less hindered carbon atom in the epoxy ring and opens the epoxide ring, which forms the metal coordinated bromo-alkoxide, and considered as rate-limiting step.^2,69^ In the following step, the primary amine groups of 3-aminopropyltrimethoxysilane-grafted ZT–PFs polarize the CO_2_ molecule to create a carbamate, and the electrophilic carbon of the incoming CO_2_ forms a tetrel bond with the negatively charged oxygen of metal-bound bromo-alkoxide. The insertion of a C–O bond of CO_2_ between the (Zr and Ti)–O(bromo-alkoxide) link results in the production of a metal carbonate intermediate, which has been claimed to be a more thermodynamically preferred phase.^70^ The –NH_2_ groups that are present in the crosslinked ZT–APFs structure may be rotated, which would further enhance this interaction. Finally, the metal carbonate species undergoes ring-closing, resulting in the elimination of bromide ions. At the same time, the cyclic carbonate is removed from the metal center, and the active catalyst is regenerated. The presence of both the Lewis acidic (Zr and Ti) centers and the N basic moiety in 3-aminopropyltrimethoxysilane-grafted ZT–PFs would make Br@ZT–APF an attractive candidate for the CO_2_ cycloaddition process. As a result, the oxygen anion from SO attacks the carbamate carbon, which converts into the corresponding cyclic carbonate (SC), as proved by cycloaddition with regeneration of the catalyst. According to the proposed mechanism illustrated in Scheme 2, the inclusion of tertiary amine results in a beneficial synergistic effect that improves catalyst performance. Additionally, the presence of halide anion increased nucleophilicity and leaving ability, with better leaving groups giving the most activity. This demonstrated the significance of nucleophilic attack on the epoxide C atom in the progression of the reaction.^23,71^

**Fig. 10:**
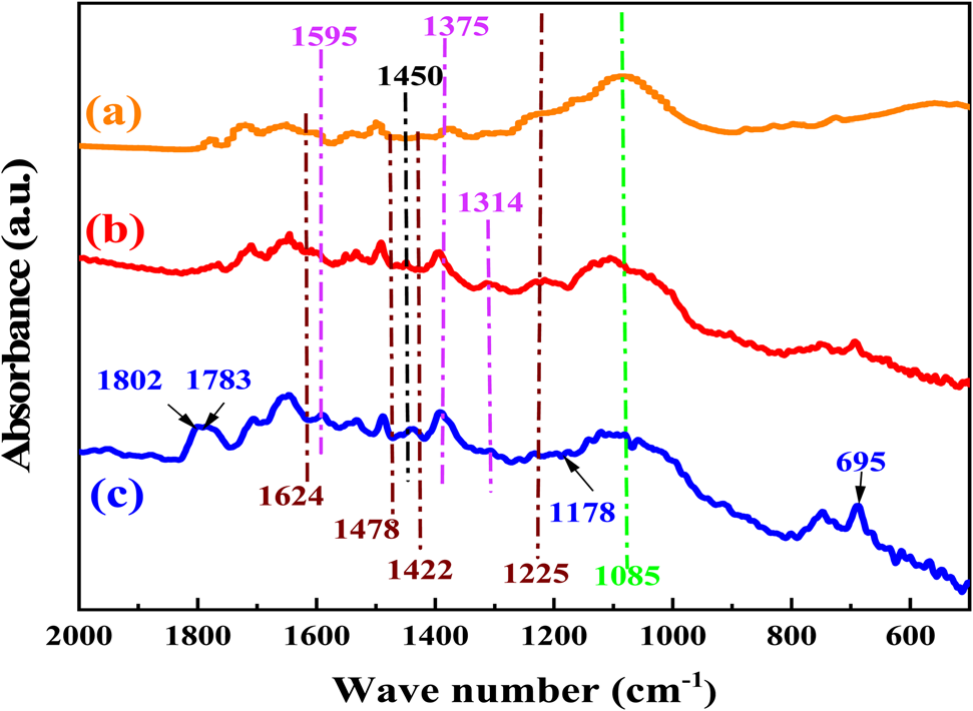
The FTIR spectra of the Br@ZT–APF. (a) Before reaction, (b) after reaction, (c) adsorption of catalyst with CO_2_.

To prove the reaction mechanism, the sites for CO_2_ activation by the catalyst were studied using the FT-IR spectrum (Fig. 10). After the catalyst was exposed to CO_2_ under optimum reaction conditions, a number of additional transmission bands emerged. The band at 1802 and 1783 cm^−1^ are due to C=O and C–O bonds respectively.^42^ Comparing the FTIR of the used catalyst with that of the fresh catalyst allowed for additional confirmation that the carbamate anion had formed. The peaks at 1624 and 1225 cm^−1^ are assigned to bidentate bicarbonate species, b-HCO_3_^−^, whereas monodentate bicarbonate, m-HCO_3_^−^, was present at 1478 and 1422 cm^−1^ as well as the peaks at 1450 and 1430 cm^−1^ are assigned to polydentate carbonate species, p-CO_3_^2−^.^72,73^ The peaks at 1595 and 1314 cm^−1^ can be assigned to bidentate carbonate species, b-CO_3_^2−^, and the peaks at 1375 and 1355 cm^−1^ can be assigned to monodentate carbonate species, m-CO_3_^2−^.^74^ The peaks at 1565 and 1466 cm^−1^ was assigned to asymmetric and symmetric stretching vibration of the RCOO^−^.^42,75–77^ Sun *et al.*^78^ investigated the effect anchorage of CO_2_ with 15 wt% monoethanolamine (MEA). Several new bands at 1568, 1486, and 1322 cm^−1^ were tethered to COO^−^ asymmetric and symmetric stretching, and N–COO^−^ stretching vibration of the carbamate species, respectively.^79–81^

## 4. Conclusions

In this study, we developed and investigated Br@ZT–APF as a bifunctional acid–base heterogeneous catalyst for the production of cyclic carbonates from epoxides and CO_2_. The synergistic interaction between amine groups and transition metal oxides (ZrO_2_ and TiO_2_) played a crucial role in activating CO_2_ and epoxides, facilitating an efficient cycloaddition reaction. Additionally, the incorporation of catalytically active Br^−^ ions within the porous ZT–APF framework significantly enhanced catalytic performance. This technology offers a sustainable and efficient pathway for CO_2_ utilization, operating under moderate reaction conditions, including low solvent use, reduced reaction temperature, shorter reaction times, and lower pressure (CO_2_) compared to previously our work.^23^ The Br@ZT–APF catalyst demonstrated outstanding activity, achieving 100% styrene oxide (SO) conversion and >99% selectivity for styrene carbonate (SC) under optimized reaction conditions. Furthermore, excellent catalyst stability and recyclability were confirmed, with consistent performance maintained over at least five consecutive cycles. Beyond its catalytic efficiency, this study contributes to the broader goal of carbon capture and utilization (CCU) by providing a viable approach for converting CO_2_ into valuable chemical products. The ability to transform CO_2_ into cyclic carbonates aligns with global sustainability initiatives, reducing greenhouse gas emissions while supporting the production of environmentally friendly materials. Cyclic carbonates have widespread industrial applications, including their use in polymer production, battery electrolytes, and pharmaceutical intermediates, making this catalytic approach highly relevant to green chemistry and industrial sustainability. Overall, this work lays the foundation for further advancements in the design of porous heterogeneous catalysts for CO_2_ valorization. Future research could focus on scaling up the process, optimizing catalyst design, and exploring other functionalization strategies to enhance performance and expand the applicability of this technology in various CO_2_ conversion processes.

## Data availability

All data generated or analyzed during this study are included in this published article in the main manuscript and ESI.†

## Conflicts of interest

The authors declare no competing financial interest.

## Supplementary Material

RA-015-D5RA00392J-s001
