# Expression of Concern: High-efficiency acid–base catalysts: ZrO_2_, TiO_2_, amine, and Br functionalized porous polymers for CO_2_ and epoxide to cyclic carbonate conversion

**DOI:** 10.1039/d6ra90022d

**Published:** 2026-02-13

**Authors:** Abbas A. Jawad, Sura A. Ahmed, Hasan J. Al-Abedi

**Affiliations:** a Midland Refineries Company MRC/AL Daura Refinery Company/Project Management Division Baghdad Iraq abbasajd5d@gmail.com abbasajd5d@outlook.com; b Department of Chemical and Biochemical Engineering, Missouri University of Science and Technology Rolla MO 65409-1230 USA; c Midland Refineries Company MRC/AL Daura Refinery Company/Maintenance Board Baghdad Iraq

## Abstract

Expression of Concern for ‘High-efficiency acid–base catalysts: ZrO_2_, TiO_2_, amine, and Br functionalized porous polymers for CO_2_ and epoxide to cyclic carbonate conversion’ by Abbas A. Jawad *et al.*, *RSC Adv.*, 2025, **15**, 7630–7643, https://doi.org/10.1039/D5RA00392J.

The Royal Society of Chemistry is publishing this expression of concern in order to alert readers that concerns have been raised regarding the integrity of the N_2_ isotherm data in Fig. 2, the FTIR spectrum in Fig. 4b and the image data in Fig. 6.

The Royal Society of Chemistry has asked the affiliated institution (Missouri University of Science and Technology) to investigate this matter and confirm the integrity and reliability of published data.

An expression of concern will continue to be associated with the article until we receive conclusive evidence regarding the reliability of the reported data.

Laura Fisher

2nd February 2026

Executive Editor, *RSC Advances*

