# Supplementary material for: High-efficiency acid–base catalysts: ZrO2, TiO2, amine, and Br functionalized porous polymers for CO2 and epoxide to cyclic carbonate conversion
Source: RSC Adv. 2025 Mar 10;15(10):7630–43. doi: 10.1039/d5ra00392j (PMC11891868; doi:10.1039/d5ra00392j)
Supplement: RA-015-D5RA00392J-s001 [file RA-015-D5RA00392J-s001.pdf]

Supplementary Information (SI) for RSC Advances.
This journal is © The Royal Society of Chemistry 2025

Supporting Information

High-Efficiency Acid-Base Catalysts: ZrO_2, TiO_2, Amine, and Br Functionalized Porous Polymers for CO_2 and Epoxide to Cyclic Carbonate Conversion

Abbas A. Jawad[a,b*], and Sura K. Ahmed[c], and Hasan J. Al-Abedi[b]
Midland Refineries Company MRC/AL Daura Refinery Company/ Project Management Division/ Baghdad, Iraq[a]
Department of Chemical and Biochemical Engineering, Missouri University of Science and Technology, Rolla, MO 65409-1230, USA[b]

abbasajd5d@gmail.com; abbasajd5d@outlook.com

Midland Refineries Company MRC/ AL Daura Refinery Company/Maintenance Board/Baghdad, Iraq[c]

Figure S1: FTIR spectra showing the styrene carbonate (SC) peaks and styrene oxide (SO).

Figure S2: The mass spectrum (MS) of the product styrene carbonate

Figure S3: ^{1}H NMR Spectra for styrene carbonate in CDCl3.

Figure S4: ^{13}C NMR spectra of Br@ZT-APF (Fresh and Used).

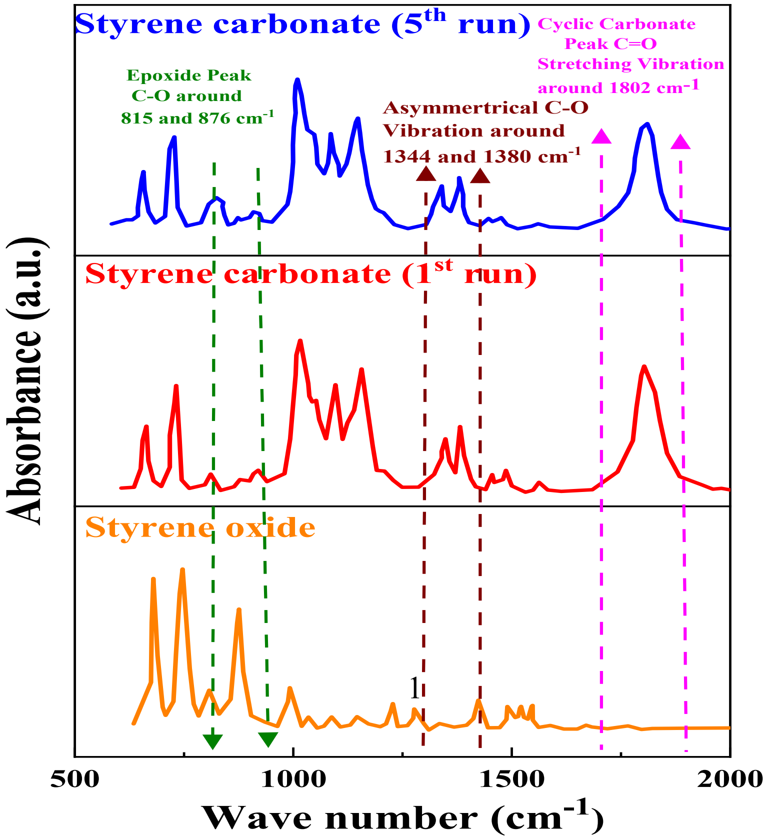

Figure S1: FTIR spectra showing the styrene carbonate (SC) peaks and styrene oxide (SO).

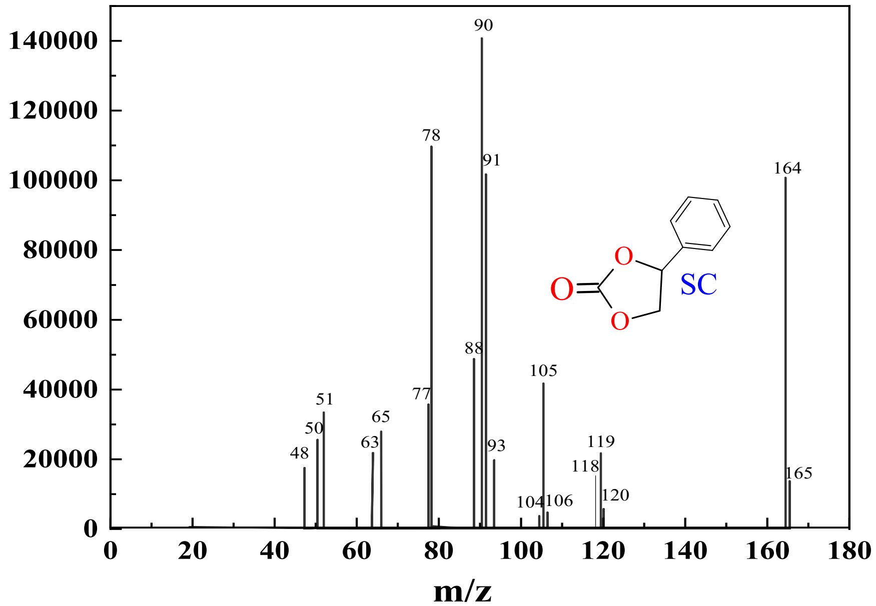

Figure S2: The mass spectrum of the product styrene carbonate.

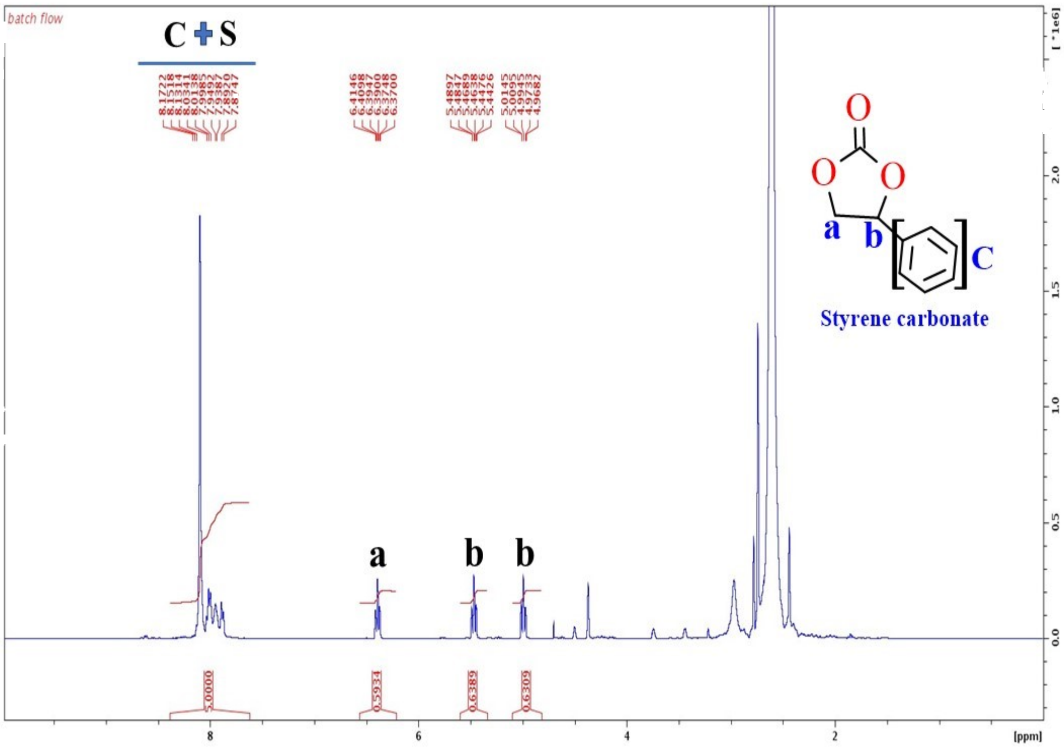

Figure S3: ^{1}H NMR Spectra for styrene carbonate in $CDCl_3$.

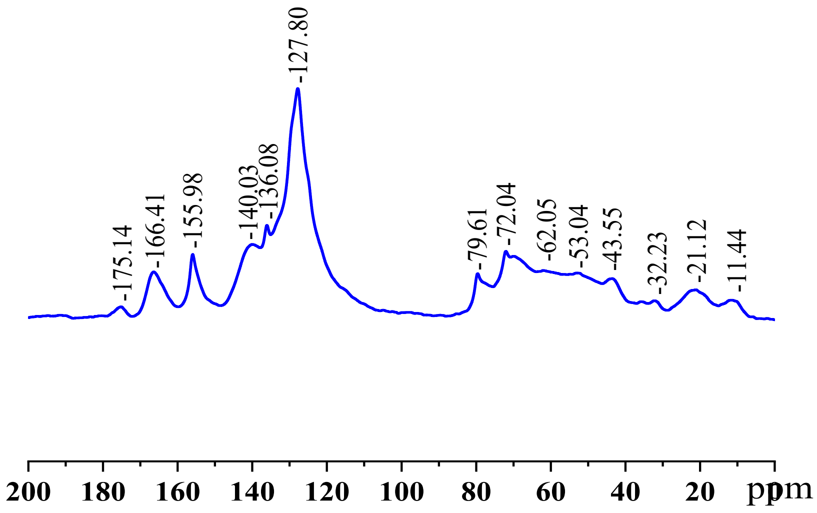

Figure S4: ^{13}C NMR spectra of Br@ZT-APF.
